# Supplementary material for: The genome sequence of the Chalk Yellow-face Bee, Hylaeus dilatatus (Kirby, 1802) (Hymenoptera: Colletidae)
Source: Wellcome Open Res. 2026 Jan 6;11:18. [Version 1] doi: 10.12688/wellcomeopenres.25424.1 (PMC12881847; doi:10.12688/wellcomeopenres.25424.1)
Supplement: Peer review history

# Reviewer report: Reviewer response for version 1

**DOI:** 10.21956/wellcomeopenres.28010.r144914

**Published:** 2026-02-06

**Authors:** Panagiotis Ioannidis

**Affiliations:** 1 Foundation for Research & Technology - Hellas, Crete, Greece

This paper reports the sequencing and assembly of the hymenopteran species Hylaeus dilatatus.

 Firstly, the sequencing coverage (185x) is pretty good; in fact, higher than whatever I've seen so far. However, despite such a good coverage and a relatively small genome (285 Mbp), the final assembly contains 798 scaffolds, which are a lot! Especially given that the expected number of chromosomes is 12. Only 82.9% of the haplotype 1 is assigned to one of these 12 chromosome-level scaffolds. In contrast, this number in most other genome assemblies I have reviewed so far is >99%.

 All other quality metrics provided by the authors are very good; QV score = 60, k-mer completeness = 98.44%, BUSCO = 98.9%.

 As a summary I am expecting that the authors make a comment on the possible causes of such a fragmented assembly. The blob plot (Figure 6) is a very nice summary in this respect; apart from the large scaffolds near the center of the plot, there is a "clould" of small scaffolds at the right (i.e. of higher %GC) and also another, smaller "cloud" at the left (i.e. of lower %GC). Such a figure cannot be presented without a comment.

 I'm not saying that such a genome shouldn't be indexed. I'm just saying that, at the very least, the authors should state that their assembly is fragmented; more fragmented than it should be, to be precise. I appreciate the effort on both the side of the authors, as well as on the side of the journal, but such findings shouldn't be left without comment. I appreciate that there a particular format, but I would expect a sentence or two about the findings.

Are sufficient details of methods and materials provided to allow replication by others?

Yes

Is the rationale for creating the dataset(s) clearly described?

Yes

Are the datasets clearly presented in a useable and accessible format?

Partly

Are the protocols appropriate and is the work technically sound?

Yes

Reviewer Expertise:

insect genomics, bionformatics

I confirm that I have read this submission and believe that I have an appropriate level of expertise to confirm that it is of an acceptable scientific standard, however I have significant reservations, as outlined above.

# Reviewer report: Reviewer response for version 1

**DOI:** 10.21956/wellcomeopenres.28010.r144241

**Published:** 2026-02-03

**Authors:** Rick Masonbrink

**Affiliations:** 1 Iowa State University, Ames, Iowa, USA

This is a significant contribution to the diversity of Hymenoptera genomes available. They’ve assembled a high-quality phased genome at chromosomal resolution using PacBio and Hi-C data. Every quality metric used points to this genome being high quality, though there are some minor issues that need to be addressed.

 Photograph 1 does not seem to be of sufficient quality and hides a large part of the external characteristics.  

 The TC buffer used in the Hi-C sample preparation needs to be written out.

 Table 5 should be referenced in the methods.

 The repeat content estimation by genomescope may be misleading as it also contains PCR/Sequencing errors. May be important to mention this caveat.

 Under Assembly statistics the two phased genomes are described differently, with one being chromosomal and one being scaffolded.  It appears to me that both have been assigned to pseudomolecules and are thus both chromosomal.  The sentence after this does inform the reader of which genome the authors are referring to.

 Figure 3 seems odd. The visible Hi-C signal indicates the genome size is around ~270Mb, with a large section shown in gray. Is this gray area comprised of exceedingly small contigs? Did the authors not perform small contig elimination?  How did the authors decide that this species has 12 chromosomes? If there is no external evidence, like a chromosome squash, then it appears that some of these chromosomes are sharing the same sequences between centromere and telomere. This is unlikely and may indicate some scaffolding errors, unless this species has telocentric/acrocentric chromosomes.

 There is an incongruity with the BUSCO version and the database used.  BUSCO v6 was released with the hymenoptera_odb12, but the text says BUSCO v6 was used with hymenoptera_odb10.  So, was Busco v6 used on an older database?

 What BLAST database was used for Blobtools? This seems like an extraordinary number of unannotated contigs.

 Figure 4 does not appear to have a red line.

 Altogether this submission warrants indexing after these minor details are investigated, as it is of high value to the scientific community. 

 Rick Masonbrink

Are sufficient details of methods and materials provided to allow replication by others?

Partly

Is the rationale for creating the dataset(s) clearly described?

Yes

Are the datasets clearly presented in a useable and accessible format?

Yes

Are the protocols appropriate and is the work technically sound?

Yes

Reviewer Expertise:

bioinformatics; genome assembly, gene annotation, Hi-C, cytology, molecular biology

I confirm that I have read this submission and believe that I have an appropriate level of expertise to confirm that it is of an acceptable scientific standard, however I have significant reservations, as outlined above.

# Reviewer report: Reviewer response for version 1

**DOI:** 10.21956/wellcomeopenres.28010.r144909

**Published:** 2026-01-21

**Authors:** Fatih Dikmen

**Affiliations:** 1 Istanbul University, Istanbul, Turkey

The manuscript presents a phased, chromosome-level genome assembly for the yellow-faced bee, Hylaeus dilatatus. The study is a significant and timely contribution to the field of insect genomics, providing a high-quality genomic resource for an important pollinator genus with unique biological characteristics.

 Hylaeus bees are particularly interesting from an evolutionary standpoint as they lack external pollen-carrying structures (scopa), instead transporting pollen internally. A high-quality reference genome for a representative species like H. dilatatus provides an essential foundation for investigating the genetic underpinnings of this and other unique adaptive traits.

 The technical quality of the genome assembly is good. The combination of PacBio long-read sequencing, providing deep coverage of approximately 185×, and Hi-C data for scaffolding has proven highly effective. The resulting quality metrics are fair. The assembly boasts a combined QV score of 60.0, which translates to an extremely low error rate, indicating a high-fidelity genomic sequence. This is further supported by the high k-mer completeness of 98.44%.

 From a functional perspective, the genome is remarkably complete. The BUSCO analysis, identifying approximately 99% of the expected single-copy orthologs from the Hymenoptera database, confirms that the assembly contains a near-complete set of genes.

 In conclusion, the authors have produced a gold-standard genomic resource for an ecologically and evolutionarily significant bee species. The methodology is sound, the results are robustly validated, and the resulting assembly is of the highest quality. This work will be good groundbase for future studies on pollinator genetics, the evolution of social behavior, and the conservation of this vital insect group. The manuscript is well-written, and the data presented clearly supports the authors' claims of a high-quality assembly. It is a valuable addition to the scientific literature.

Are sufficient details of methods and materials provided to allow replication by others?

Yes

Is the rationale for creating the dataset(s) clearly described?

Yes

Are the datasets clearly presented in a useable and accessible format?

Yes

Are the protocols appropriate and is the work technically sound?

Yes

Reviewer Expertise:

Genetics, Taxonomy, Hymenoptera,

I confirm that I have read this submission and believe that I have an appropriate level of expertise to confirm that it is of an acceptable scientific standard.
